# Characterization of High-Risk Oncogenic Human Papillomavirus Genotypes in Histologically Confirmed Ear, Nose and Throat (Ent) Cancers in Burkina Faso

**DOI:** 10.31557/APJCP.2019.20.11.3429

**Published:** 2019

**Authors:** Maïmouna Ilboudo, Théodora Mahoukèdè Zohoncon, Esther Mah Alima Traore, Ina Marie Angèle Traore, Ali Kande, Florencia W Djigma, Dorcas Obiri-Yeboah, Yvette Marie Chantal Gyebre, Jacques Simpore

**Affiliations:** 1 *Laboratory of Molecular Biology and Genetics (LABIOGENE), University Joseph KI-ZERBO, 03 BP 7021 Ouagadougou 03, *; 2 *Pietro Annigoni Biomolecular Research Center (CERBA), 01 BP 364 Ouagadougou 01, *; 4 *University Joseph KI-ZERBO, 03 BP 7021 Ouagadougou 03; University Hospital Yalgado OUEDRAOGO, Burkina Faso, *; 3 *University of Cape Coast, School of Medical Sciences, Department of Microbiology and Immunology, University Post Office, Ghana. *

**Keywords:** Human papillomavirus (HPV), high-risk HPV, carcinoma, real-time PCR, ENT, Burkina Faso

## Abstract

**Background:**

High-risk human papillomavirus (HPV-HR) infections are responsible for 99.99% of cervico-uterine cancers and 50% of carcinomas of the oropharynx.

**Objective:**

To characterize high-risk HPV genotypes (HPV-HR) in histologically confirmed ear, nose and throat (ENT) cancers in Ouagadougou.

**Methods:**

One hundred and twenty-eight archived tissues from the ENT sphere, obtained over the last ten years (2007 to 2017) and histologically diagnosed in anatomy and pathology-cytology laboratories in Ouagadougou were included. These tissues were dewaxed with xylene; HPV DNA extraction was performed and HPV-HR were researched by real-time multiplex PCR.

**Results:**

Among the fourteen HPV-HR genotypes tested for, seven were identified. The prevalence of HPV-HR infection was 15.6%. The most common genotypes were: HPV56 (45%) and HPV33 (20%). Squamous cell carcinomas accounted for 75% of cases, followed by lymphomas for 10%. The age range was between 5 and 80 years.

**Conclusion:**

The results show the involvement of a diversity of HPV-HR genotypes and a high frequency of HPV56 and HPV33 in ENT cancers in Ouagadougou, Burkina Faso. The appropriate HPV vaccination will considerably reduce the number of these cancers.

## Introduction

Ear, Nose and Throat (ENT) cancers are common worldwide. These cancers are most often secondary to alcohol and tobacco abuse. However, several studies more than thirty years ago, have shown the role of HPV in the genesis of these cancers (Löning et al., 1985; Chang et al., 1991). The HPV most commonly involved are HPV 16, 18, 31, 33, 35, 39, 45, 51, 52, 56, 58, 58, 59, 66 and 68 (Gillison et al., 2012). Induced HPV cancers are most often observed in younger subjects than those with carcinomas secondary to alcohol and tobacco intoxication. In general, human papillomavirus has a tropism for skin and mucosal epithelial cells but can survive freely in the environment for several months, which contributes significantly to its virulence. Transmission can occur through indirect contact with contaminated surfaces; through urogenital sex or during child delivery. 

In 95% of cases, ENT cancers due to HPV are very homogeneous carcinomas histologically. They are made up of squamous cell carcinomas whose prognosis remains generally severe. The advanced stages of ENT cancers are dominated by the risk of recurrence, local and/or regional, cervical lymph node cancer. The treatment of these types of cancers involves therapeutic modalities, combining local and lymph node surgery, radiotherapy and chemotherapy (Lacau et al., 2010). At all stages, oropharyngeal carcinomas are serious cancers with an estimated 40% survival at two years without recurrence (Bossard et al., 2007), mainly due to the risk of local and/or regional recurrence.

Globally, cancers are among the leading causes of morbidity and mortality. ENT cancers are ranked 5th of carcinomas in humans, in the world (Auperin et al., 2011). The prevalence of HPV-related oropharyngeal cancers increased from 16% to 72% between 1984 and 2004 in the United States. The increase in the incidence of oropharyngeal cancers is related to HPV (Chaturvedi et al., 2011). In Africa, cancers already account for between 10 and 20% of pathologies on the continent and cancer mortality is proportionally higher than elsewhere in the world. 

The prevention of HPV (+) ENT cancers necessarily involves raising awareness about hygiene and sexual behavior, but also vaccination. HPV vaccination is likely to significantly reduce the number of these cancers worldwide. Recently, two prophylactic vaccines against human papillomavirus, based on immunogenicity, have been developed. These are Cervarix (Silbermann and Launay, 2007), a bivalent vaccine that is effective against HPV genotypes 16 and 18, and Gardasil 9, which combines five HPV-HR genotypes (16, 18, 31, 33, 45, 52, 58) and two HPV-BR genotypes (6-11). These vaccines are only administered from the age of 09 years and do not cover all 14 HPV-HR genotypes. The identification of the different HPV genotypes in ENT cancers could improve the short-term prognosis and directly influence therapeutic modalities. 

In Burkina Faso, cancer of the ENT and cervico-facial sphere is relatively frequent and represents 24.5% of this entity (Ouoba et al., 1997). Several studies have already been done on HPV in different populations of women with or without cervical lesions in Burkina Faso (Zohoncon et al., 2013; Traore et al., 2016; Ouedraogo et al., 2018). No study has yet been conducted in Burkina Faso on the involvement of HPV in ENT cancers. In view of the increasing number of ENT tumors induced by HPV in the world, it therefore seemed important to us to study the involvement of HPV- HR genotypes in ENT cancers in Burkina Faso. This, in order to consider prevention strategies, of which HPV vaccination is an important part.

## Materials and Methods

*Type and population of study*

The study was led in the city of Ouagadougou, the political capital of Burkina Faso, and we conducted a descriptive cross-sectional study with retrospective data collection from 2007 to 2017. We consulted the anatomy and pathology cytology registers of the Yalgado Ouedraogo University Hospital Center (CHUYO), the Shiphra Clinic, the Sandof Clinic and the Philadelphia Clinic in Ouagadougou, Burkina Faso. The samples were constituted by histologically confirmed ENT cancer tissues that had been treated and stored in paraffin between 2007 and 2017.

*Sampling and data collection*

These paraffin blocks containing a biopsy piece were cut with a microtome in the pathology and cytology anatomy laboratory at the CHU-YO to obtain five sections about 20μm in thickness. Then these tissues collected in Eppendorf tubes were sent to the Laboratory of Molecular Biology and Molecular Genetics (LABIOGENE) for molecular analyses.

*Human papillomavirus research*

*Dewaxing of paraffin fabric*

DNA extraction was performed with the NORGEN FFPE DNA Purification kit according to the protocol provided by the manufacturer. But before this step we proceeded to the dewaxing of the tissues with xylene.

*Dewaxing of samples*

1mL of xylene was added to the sample, vortexed and incubated at 50°C for 10 minutes and centrifuged at 14,000 RPM for 2 minutes. After centrifugation, the supernatant was removed and the step was repeated with xylene for a second time. After the xylene step, we added 1 ml of absolute ethanol to the sample, which we then vortexed and centrifuged at 14,000 RPM for 2 minutes. The supernatant was discarded and the ethanol step was repeated for a second time.

*Extraction of HPV DNA after dewaxing*

After the xylene dewaxing step, we used the NORGEN kit for extraction of HPV DNA according to the manufacturer’s protocol. The steps of this extraction were cell lysis followed by heating by incubation at 50°C for 1 hour then 90°C for 1 hour to release the genetic material, precipitation of DNA with absolute ethanol, binding of DNA to the columns and finally the three series of column washing and DNA eluting. 

*Real-time PCR for the detection of high-risk HPV*

PCR amplification of the DNA extracted from the samples in this study was performed to look for human papillomaviruses. PCR was performed on the SaCycler-96 Real Time PCR v.7.3 equipment (Sacace Biotechnologie, Italy) with the “High Risk Typing Real-TM” kit from SACACE biotechnologies^®^. This kit detects fourteen high-risk HPV genotypes (HPV 16, 18, 31, 33, 35, 39, 45, 51, 52, 52, 56, 58, 59, 66 and 68”. The PCR was performed according to the protocol described by the manufacturer. The PCR program used was as follows: 1 cycle at 95°C for 15 minutes; 5 cycles of 95°C for 05 second, 60°C for 20 second and 72°C for 15 second; 40 cycles of 95°C for 05 second, 60°C for 30 second and 72°C for 15 second.

*Statistical analysis*

The statistical analysis were performed using IBM SPSS statistics 20 software and the chi-square test was used to compare the results. The results were considered significant for a p-value of less than 5%.

*Ethics and consent*

This study was approved by the Research Ethics Committee of Saint Camille Hospital Ref. 2017/CERBA/II-24/0019 du 24-02-2017 in Burkina Faso. We conducted a descriptive cross-sectional study with retrospective data collection from 2007 to 2017 and the samples were constituted by paraffin blocks containing a biopsy. The confidentiality and anonymity of the information collected has been respected. 

## Results

*Characteristics of ENT cancer samples from 2007-2017*

Patients had mainly reported to the clinic due to significant pain in the buccal cavity, and sometimes bloody sores. The site of the lesion was the larynx, pharynx, endolarynx and palate. The macroscopic aspect of the lesions was in the form of budding, ulceration with signs of local and regional extension (adenopathy). Additional examinations supporting the diagnosis or extension were also taken into account. 

**Table 1:** Carrying HPV-HR According to the Location of the Tumor

Location of the tumor	Oral	Nasal	Ear	Totaln (%)	P value
HPV-HR-	96 (84.85)	9 (75)	3 (100)	108 (84,37)	
					0.722
HPV-HR+	17 (15.4)	3 (25)	0 (0)	20 (15,63)	
					
Total	113 (88,28)	12 (9,3)	3(2,3)	128 (100)	

**Figure 1:**
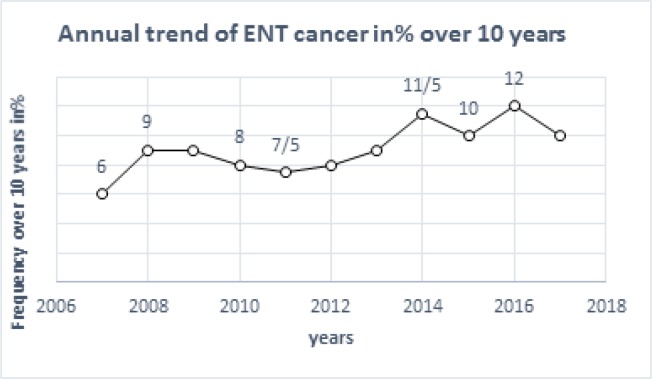
Distribution of ENT Cancer Cases by Year and Gender from 2007 to 2017

**Figure 2:**
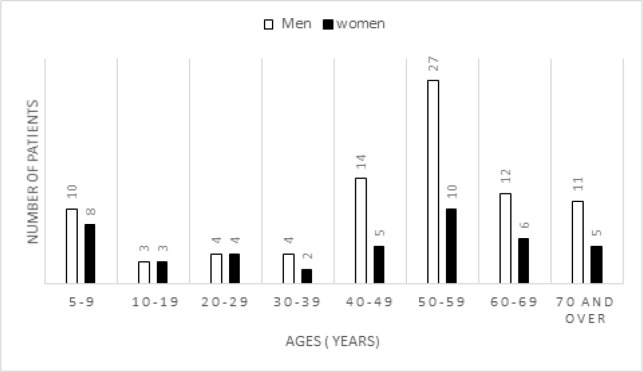
Distribution of ENT Cancer Cases by Age and Gender

**Table 2:** Localization of ENT Cancers According to the Prevalence of HPV-HR Genotypes

Localization of ENT cancers	HPV genotype	HR-HPV Prevalence
Larynx (27/128)	HPV16	5%
	HPV16-33	5%
	HPV33	5%
	HPV45	5%
Palate (27/128)	HPV33	5%
	HPV52	5%
	HPV56	20%
Maxillary (21/128)	HPV33	5%
	HPV18-33	5%
	HPV56	10%
Nasal (10/128)	HPV18	5%
	HPV33	5%
Pharynx (16/128)	HPV56	10%
Tongue (08/128)	HPV39	5%
Amygdala (1/128)	HPV56	5%
Gums (10/128)	-	-
cheek (4/128)	-	-
Lips (1/128)	-	-
Ear (3/128)	-	-

**Table 3:** Distribution of HPV-HR Genotypes by Histological Type of ENT Cancer

Number of ENT cancer types	HR-HPV genotype	Prevalence
	HPV56	40%
CARCINOMAS (97/128)	HPV33	20%
	HPV16	5%
	HPV16-33	5%
	HPV18	5%
	HPV39	5%
	HPV45	5%
	HPV52	5%
LYMPHOMAS (14/128)	HPV56	5%
MELANOMAS (4/128)	HPV33-18	5%
SARCOMAS (7/128)	-	-
CYLINDROMES (06/128)	-	-

**Figure 3:**
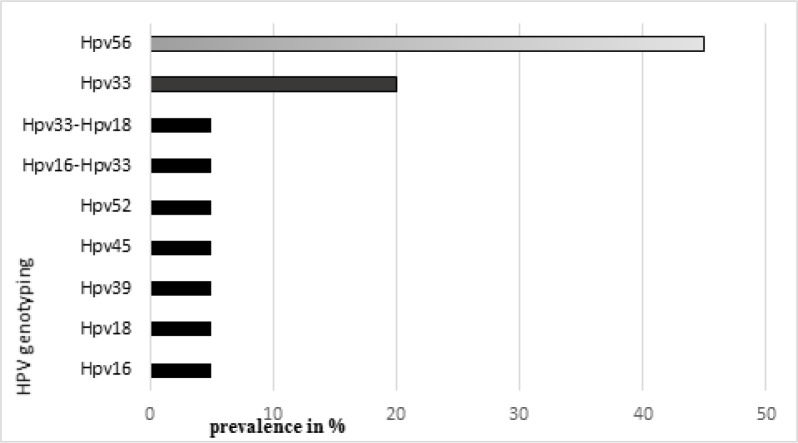
Prevalence of HPV-HR Genotypes in Samples

*Etiology and epidemiology of ENT cancer cases from 2007-2017*

From 2007 to 2017, a total of 128 histologically confirmed cases of ENT cancer were diagnosed in the anatomy and cytology pathology laboratories of the CHU-YO, Shiphra Clinic, Sandof Clinic and Philadelphia Clinic in Burkina Faso (Figure1). These patients actually came from all the provinces of Burkina Faso. Histologically, the samples taken in our study showed a malignant tumor proliferation, suggestive of carcinomatous degeneration.

The clinical records of patients does not mention all the information on the risk factors. But for those who mentioned it, it was noted that the main risk factors were tobacco, alcohol, and lack of oral hygiene, the presence of tartar, caries, dental snags and chronic ENT infections. This retrospective study focused on 128 cases of ENT carcinomas in a population aged between 05 and 80 years with an average age of 41 years ±19 years. The tissue blocks collected were from 85 male and 43 female patients, representing a gender ratio of 1.97. 

The distribution of cases by age and gender is summarized in Figure 2 (P=0.920). The most affected age group was 40-59 years (41%) and no statistically significant difference was obtained in the distribution of HPV by gender (male/female) (P=0.820).

Clinically, 50% of our patients came for a late visit. The most common symptoms encountered were: a large budding and/or infiltrating tumor, an ulcer-budding tumor, and tumor bleeding on contact. The most frequent reason for consultation was respiratory distress, requiring tracheotomy, emergency laryngectomy or almost total chronic dysphagia or dysphonia. 

*Prevalence of HPV infection in ENT cancers*

In this study, all 128 biopsy tissue blocks, fixed to formalin and paraffin-embedded, histologically tested positive for ENT cancers gave a valid PCR result. The results obtained showed that 15.6% (20/128) of the tissue blocks tested were infected with HPV-HR. Among these samples carrying HPV-HR, the predominant genotype was HPV56 with a prevalence of 45% followed by HPV33 (20%); co-infections HPV33-HPV18 and HPV16-HPV33 each represented 5% (Figure 3).

*Prevalence of HPV infection by tumor location*

According to the location of the tumor, oral cancers were the most frequent with 88.3% of cases (113/128), followed by nasal cancer with 9.4% (12/128) and ear cancer with 2.3% (3/128). The prevalence of HPV-HR was relatively higher in oral than nasal cancer, while no HPV-HR was found in ear cancer (Table 1). 

In oral cancer, the larynx (27/128) was in the lead followed by the palate (27/128), and then maxillae (21/128). However, in terms of genotypic variability, the larynx was in the lead with a carriage of four different genotypes of HPV (HPV16, 45, 33.52) followed by the palate and maxillae that carry the same number of genotypes. On the genotypic aspect, the palate was in lead (20% HPV56) followed by the pharynx (10% HPV56). In this study, cancers of the cheek (4/128), ear canal (3/128), gums (10/128) and labia (1/128) showed no genotypic carriage of HPV (Table 2). 

*Genotyping of HPV-HR type according to the histological diagnosed ENT cancers*

In this study, ENT cancers were largely dominated by squamous cell carcinomas (75.8%). Malignant lymphomas present in 10.9% of cases were Burkitt’s tumors, Hodgkin’s disease and non-Hodgkin’s malignant lymphoma (NHL). These lymphomas were followed by sarcomas (5.5%) consisting of myosarcomas, fibrosarcomas, osteosarcomas and cylindroids (4.7%). Malignant melanoma, although having a significant impact on the mucous membranes of the ENT sphere and more particularly on the nasal cavities and sinuses, has been noted only very rarely (3.1%). According to histological type, squamous cell carcinomas had a highest HR-HPV carrying rate followed by melanomas and lymphomas. The distribution of HR-HPV is given by Table 3. No HR-HPV was found in sarcomas and cylindromes. 

## Discussion

This retrospective study, covering 128 cases of ENT cancers from 2007-2017, reports a similar overall frequency of 12.8 cases per year in Ouagadougou over a 10-year period. These data do not reflect reality because many patients do not arrive at the hospital deliberately, referred to traditional practitioners out of ignorance or for economic reasons. Nevertheless, this incidence seems significant to us, but it is lower than the 217 cases of Ouoba et al., (1997). This difference in results could be explained by the fact that these authors worked on tumors in the ENT and cervico-facial sphere dating from 1985 to 1994 and coming from the two national hospitals in Burkina Faso at that time, the ENT Department, Ouagadougou University Hospital and the Odonto-stomatology and Maxillofacial Surgery Department, Bobo Dioulasso University Hospital. Other authors reported 79 cases in the city of Ouagadougou between 2003 and 2014 (Löning et al., 1985). However, these authors only worked on oral samples from the only pathological anatomy laboratory in the CHU-YO operating in the country at that time. Our results are also lower than the 219 cases of Njimah et al., (2018) in Douala. This difference could be explained by the fact that many cases were not included in our series, especially during the collection, some biopsy parts were not found. 

In this study, the increase in the number of annual cases of ENT cancer could be explained by increase in health services, the improvement in care with the increase of the number of specialists, but also by greater attendance at hospital facilities. Indeed, before 2007, Burkina Faso had only one pathological anatomy laboratory located in CHU-YO (the country’s reference center). This number has increased to five nowadays (Yalgado Hospital, Blaise Compaoré Hospital, Shiffra Clinic, Sandof Clinic and Philadelphia Clinic), thus improving access to care. In ENT cancers, the main risk factors are tobacco and alcohol; they act by direct action but also by metabolic disturbance (Chang et al., 1991; Gillison et al., 2012; Lacau et al., 2010). However, although HPV is involved in 95% of cervical cancers, it is also found in oral cavity cancer with a relative risk of about 1.5 (Bossard et al., 2007). 

All ages are affected by ENT cancers, in this study the youngest was 05 years old and the oldest 80 years old. The most affected age group was 40 to 59 years (41%) and the average reported age was 41 years. Several authors have reported the same results (Herrero et al., 2003). Our results can be explained by the high number of patients under 10 years of age due to malignant lymphomas in general and Burkitt’s lymphoma in particular, which are neoplasms of children. Epithelial cancers are more frequent in adults and the elderly (De Camargo et al., 2010). Our study reports a male predominance of 1.97 and the most affected age group was 40-59 years (41%). Other authors have also found the same results (Gillison et al., 2012 ; Auperin et al., 2011; Chaturvedi et al., 2011; Bambara et al., 2015; Jemal et al., 2011). However, some authors have reported a female predominance (Jayalekshmi et al., 2009; Löning et al., 1985). These results could be explained by the fact that women are protected by hormonal secretions (Bambara et al., 2015). And also by the possible risk factors of tobacco and alcohol, which are predominantly male habits. In this study no statistically significant difference was obtained in the distribution of HPV by gender of patients (P=0,920). According to some authors, HPV positive Oropharyngeal cancer may affect men more heavily than women in different populations for reasons that are unclear (Combes et al., 2014). However, in this study, the small size of sample (128), not allow us to generalize our results on the prevalence of HPV-HR genotypes according to gender (male/female) in Burkina Faso. 

This study reports a general prevalence of 15.6% of HPV-HR (20/128) of which 45% of HPV56 followed by 20% of HPV33. The other genotypes HPV16, HPV18, HPV39, HPV45, HPV52, and the co-infections HPV33-HPV18 and HPV16-HPV33 each represented 5% of the cases. The absence of other HPV-HR genotypes in this study would probably be due to the sample size (128 cases) and the SACACE biotechnologies^®^ “High Risk Typing Real-TM” HPV kit, which can only detect 14 HPV genotypes (HPV 16, 18, 31, 33, 33, 35, 39, 45, 51, 52, 56, 58, 59, 66 and 68). In some authors, the overall reported prevalence is 25% for other HPV types and 90% for HPV16 (Lawson and Blitzer, 1987). These authors worked on tumors of the head and neck. As for the HPV33, 45, 52, 56 genotypes, they have been found at least once by other authors (Langdon and Rapidis, 1979). The mode of HPV transmission, the age and individual immunity, as well as the many sexual practices, would probably explain these differences in results (D’Souza et al., 2007; Gillison et al., 2012).

Focusing on tumor localization, oral cancer was the majority (88.3%) in this study followed by nasal cancer (9.4%) and hearing cancer (2.3%). In oral cancer, the larynx was the majority followed by the maxillary. Similarly, genetic variability was high at these two sites compared to other sites; HPV 16, 45, 33.52 at the larynx and HPV 56, 33.18-33 in the maxillary. But the highest prevalence by genotype is carried by the palate (20% of HPV56) followed by the pharynx (10% of HPV56). Topographically, the upper aero-digestive pathways were the preferred site for ENT and cervico-facial cancers, with 70.78% of cases in Douala (Njimah et al., 2018), while in Togo, the salivary gland cancers were the most prevalent (28.2%) followed by larynx cancers (24%) (Darré et al., 2015). The larynx cancers are usually caused by smoking. In 2004, they represented 1.5% of all cancers (Peng et al., 2004). Some authors report a predominance of oral localization to Madagascar (Randriamanovontsoa et al., 2015; Rakotoarivony et al., 2014) and oropharyngeal in Canada (Delouya et al., 2012). In some authors, localization is function of gender (Bambara et al., 2015). These authors report that among men, cancers of the tongue and palate predominated, while cancers of the lip and cheek were most common among women. In many countries, the most common oral tumor location is tongue (Sutandyo et al., 2014). In several studies, the HPV16 genotype is majority in cancers of the upper aero-digestive tract, followed by the HPV18 genotype (Si-Mohamed et al., 2012). The involvement of HPV appears vary according to the lesion site and geographical origin of the patients. 

In the present histological study, squamous cell carcinomas (97/128) largely dominated at 75.8%, with 40% of HPV56 genotypes and 20% of HPV33, malignant lymphomas came in second position (10.9% of cases) with 5% of HPV56 genotypes followed by sarcoma (5.5% of cases) and cylindrome (4.7%). Generally histological point of view, several authors have reported similar results (Amana et al., 2014; Darré et al., 2015; Amana et al., 2016). In Togo, squamous cell carcinomas dominated at 61.4% followed by non-Hodgkin’s lymphoma at 23.2% (Amana et al., 2016). Among malignant lymphomas, Burkitt’s tumor represented a strong contingent in many authors in the sub-region (Madani et al., 2005). On the genotypic level, some authors report a higher prevalence of HPV16 at 65.2% in squamous cell carcinomas in ENT compared to our results (Gavid et al., 2014). In this study, the late diagnosis of ENT cancers may also explain these results. This diagnosis makes it difficult to manage. It is therefore crucial to focus on prophylactic vaccination in the control of these ENT cancers. 

In Burkina Faso, a developing country, ENT cancers are relatively common, diagnosed at any age with a high fatality rate. In this study, the most frequent locations were the oral cavity, larynx and palate. The histological nature of cancers was largely dominated by squamous cell carcinoma, followed by lymphomas and sarcomas. Genotypes HPV 16 and HPV 18 included in the pilot trial in Burkina Faso, which are frequently associated with cancers worldwide, have not been found among the most frequent oncogenic HPVs (HPV56 and HPV33). Hence the need to take into account the genotypes circulating in the vaccine strategy in order to improve case management. 
